# Bispecific costimulatory molecules for activation of tumor-killing lymphocytes

**DOI:** 10.1186/1475-2867-4-S1-S1

**Published:** 2004-07-01

**Authors:** R Weth, M Biburger, W Wels

**Affiliations:** 1 Chemotherapeutisches Forschungsinstitut Georg-Speyer-Haus, Frankfurt am Main, Germany

## 

The receptor tyrosine kinase ErbB2 (HER2) is overexpressed in multiple human tumors of epithelial origin. High ErbB2 expression is functionally involved in tumorigenesis and correlates with poor clinical prognosis. For immunotherapy of ErbB2 expressing tumors, we developed a strategy to supply the tumor cells with costimulatory activity. A bispecific fusion protein was constructed (BIg5), containing the IgV-like domain of huCD86, the CH2/CH3 domain of huIgG1 and the ErbB2-specific single chain antibody fragment scFv(FRP5). A similar fusion protein lacking the CD86 domain (Ig5) was used as a control. Upon binding of BIg5 to ErbB2 on tumor cells, these cells display CD86 on their surface and thus can deliver costimulatory signals for T-cell activation. In addition, NK cells could be activated by CD86 binding to CD28. BIg5 is secreted by eukaryotic cells as a homodimer with increased stability compared to monomers and possibly enhanced costimulatory activity due to crosslinking of CD28 on effector cells. By FACS analysis, specific binding of the scFv(FRP5) domain to ErbB2 as well as CD86 IgV binding to CTLA-4 could be demonstrated. Together with anti-CD3 antibody, BIg5 stimulates proliferation of human CD2-purified lymphocytes *in vitro*. After binding to ErbB2 on murine Renca-lacZ/ErbB2 tumor cells, about 50% of initially bound BIg5 is still present on the cell surface after 4 hours. For delivery of chimeric fusion proteins *in vivo*, we used syngeneic, stably transfected HC11 mammary epithelial cells continuously secreting the proteins. Inoculation of these bystander cells close to subcutaneously growing Renca-lacZ/ErbB2 tumors should provide a long-lasting source to achieve high local concentrations of BIg5 at the tumor site. *In vivo *HC11-BIg5 cells proved to be non-tumorigenic and secreted BIg5 for several weeks, causing a strong anti-BIg5 antibody response. Treatment of established Renca-lacZ/ErbB2 or ErbB2-negative Renca-lacZ tumors by peritumoral inoculation of either HC11-BIg5 or HC11-Ig5 cells led to rejection of all Renca-lacZ/ErbB2, but none of the Renca-lacZ tumors. HC11neo control cells had no effect on tumor growth. Rejection of ErbB2^+ ^tumors led to long-term protection also against subsequent challenge with intravenously injected ErbB2^- ^tumor cells. Intraperitoneal injection of bystander cells secreting the fusion proteins did not lead to tumor regression suggesting that high local concentrations at the tumor site are necessary to target ErbB2 on tumor cells and to overcome elimination of BIg5 or Ig5 by neutralizing antibodies. The CD86 IgV domain of BIg5 did not play a major role in the observed antitumoral immune response suggesting NK-cell mediated ADCC as the initial effector mechanism followed by activation of tumor specific T cells. Targeting of ErbB2 on tumor cells with antibody fusion proteins that interact specifically with the host immune system could be an efficient and specific approach for therapy of solid ErbB2^+ ^tumors.

